# Association of Manganese Biomarker Concentrations with Blood Pressure and Kidney Parameters among Healthy Adolescents: NHANES 2013–2018

**DOI:** 10.3390/children8100846

**Published:** 2021-09-25

**Authors:** Maria D. Politis, Jacob C. Freedman, Erin N. Haynes, Alison P. Sanders

**Affiliations:** 1 Department of Pediatrics, Icahn School of Medicine at Mount Sinai, New York, NY 10029, USA; maria.politis@mssm.edu; 2 Department of Environmental Medicine and Public Health, Icahn School of Medicine at Mount Sinai, New York, NY 10029, USA; jacob.freedman@icahn.mssm.edu; 3 Department of Epidemiology Preventative Medicine and Environmental Health, College of Public Health, University of Kentucky, Lexington, KY 40506, USA; erin.haynes@uky.edu; 4 Department of Environmental and Occupational Health, University of Pittsburgh, Pittsburgh, PA 15260, USA

**Keywords:** manganese, kidney parameters, estimated glomerular filtration rate, blood pressure, blood urea nitrogen

## Abstract

Deficiency or excess exposure to manganese (Mn), an essential mineral, may have potentially adverse health effects. The kidneys are a major organ of Mn site-specific toxicity because of their unique role in filtration, metabolism, and excretion of xenobiotics. We hypothesized that Mn concentrations were associated with poorer blood pressure (BP) and kidney parameters such as estimated glomerular filtration rate (eGFR), blood urea nitrogen (BUN), and albumin creatinine ratio (ACR). We conducted a cross-sectional analysis of 1931 healthy U.S. adolescents aged 12–19 years participating in National Health and Nutrition Examination Survey cycles 2013–2014, 2015–2016, and 2017–2018. Blood and urine Mn concentrations were measured using inductively coupled plasma mass spectrometry. Systolic and diastolic BP were calculated as the average of available readings. eGFR was calculated from serum creatinine using the Bedside Schwartz equation. We performed multiple linear regression, adjusting for age, sex, body mass index, race/ethnicity, and poverty income ratio. We observed null relationships between blood Mn concentrations with eGFR, ACR, BUN, and BP. In a subset of 691 participants, we observed that a 10-fold increase in urine Mn was associated with a 16.4 mL/min higher eGFR (95% Confidence Interval: 11.1, 21.7). These exploratory findings should be interpreted cautiously and warrant investigation in longitudinal studies.

## 1. Introduction

Manganese (Mn) is an essential nutrient required for normal human development, with critical roles in energy metabolism, protection against oxidative stress, immunological system function, and nervous system function [1,2]. Changes in body Mn status, either overexposure or insufficiency, are associated with neurological dysfunction, as well as altered neuronal physiology and cognition in humans [3]. Among children, Mn is an established neurotoxicant, but much less is known about nephrotoxic effects during critical periods of kidney function maturation from infancy to late adolescence.

While evidence supports Mn nephrotoxicity at high doses [4,5], low-dose Mn exposure may be protective against preeclampsia or kidney disease [6,7]. Additionally, subgroups that may be particularly susceptible to adverse effects of Mn exposure include newborns and children, as well as individuals with liver conditions or iron deficiency. Exposure to Mn by ingestion or inhalation among children poses higher risks compared to adults, due to metabolic differences in absorption and elimination [8]. For example, the intestinal absorption rate of ingested Mn is higher in children and the high demand of iron linked to growth can further enhance the absorption of ingested Mn [8]. The Mn excretion rate is lower in younger people compared to adults, as the ratio of inhaled air/body weight is substantially higher and due to the less-developed biliary excretion mechanisms, as observed in neonatal animal studies [9].

Although limited by study design and challenges in exposure assessment, the evidence of adverse effects of Mn exposure on United States (U.S.) adolescents is compelling enough to warrant further research. This study aimed to investigate the potential relationship between blood and urine Mn concentrations and blood pressure and kidney parameters including estimated glomerular filtration rate (eGFR), blood urea nitrogen (BUN), and albumin creatinine ratio (ACR). The study expands upon prior research by examining the U.S. adolescent population and testing for evidence of effect modification by race/ethnicity and sex.

## 2. Materials and Methods

### 2.1. Study Design and Population

**Figure 1:**
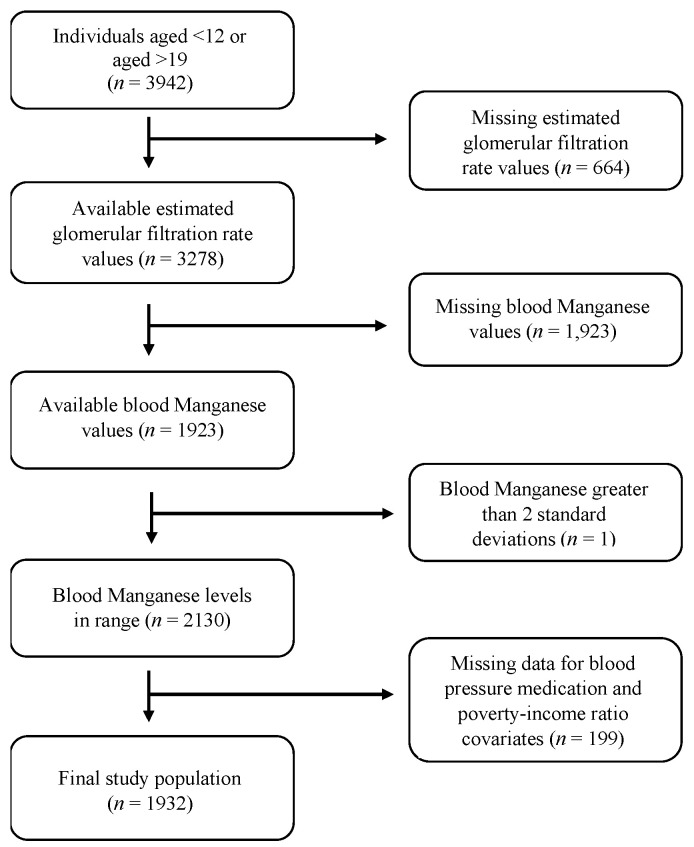
Flowchart of sample selection, National Health Examination and Nutrition Examination Survey, 2013–2018.

We used data from the National Health and Nutrition Examination Survey (NHANES), a cross-sectional survey that is nationally representative of the noninstitutionalized U.S. population. The survey procedures were approved by the National Center for Health Statistics Internal Review Board, and all participants gave informed consent. This study was exempt from review by the Icahn School of Medicine at Mount Sinai’s (ISMMS) Institutional Review Board (#1702145; approval date 07/2021). We obtained publicly available data collected through household interviews and physical and laboratory examinations from NHANES 2013–2014, 2015–2016, and 2017–2018 for adolescents aged 12 to 19 years, for a total of 3942 participants. We excluded participants who had missing values for eGFR (*n* = 664), missing values for blood Mn measurements (*n* = 1147), blood Mn measurements outliers (*n* = 1), missing values for poverty income ratio (*n* = 195), and those who took BP medication (*n* = 4). The final analytic sample consisted of 1931 participants with available blood Mn measurements (Figure 1). In secondary analyses, we examined relationships in a subset of 691 participants with available urine Mn measurements for the NHANES 2013–2014 and 2015–2016.

### 2.2. Outcome Measurements

Serum creatinine was measured via the Jaffe rate method, an enzymatic method used to determine the concentration of creatinine in serum, plasma, or urine in which creatinine was converted to creatine under the activity of creatininase. eGFR was calculated using the original Bedside Schwartz formula as the main method of measuring eGFR:

(1) $$\mathrm{eGFR}=0.413\times \frac{\mathrm{Height}\;\left(\mathrm{cm}\right)}{\mathrm{Serum}\;\mathrm{Creatinine}\;\left(\mathrm{mg}/\mathrm{dL}\right)},$$

as recommended in conjunction with this laboratory method [10]. These three additional eGFR formulae that use serum creatinine to estimate GFR were used in separate sensitivity analyses to examine any differences in the estimates.

Three consecutive blood pressure (BP) readings were obtained after the participant rested quietly in a seated position for 5 min and once the participant’s maximum inflation level was determined; a fourth attempt was made if a BP measurement was interrupted or incomplete. An average value for both systolic (SBP) and diastolic BP (DBP) was calculated using the second and third BP readings. If one or both of the second and third BP readings were missing or had zero values, then averages of two of the available readings were used. We used the single BP reading for those participants who only had one BP reading available.

BUN was analyzed on a Beckman UniCel DxC800 Synchron (Brea, CA) by Collaborative Laboratory Services, LLC., to determine the concentration in serum or plasma by means of the enzymatic conductivity rate method. The lower limit of detection (LLOD) was 1.00 mg/dL for BUN. Detailed information on specimen collection and processing is available in the NHANES laboratory procedures manual [11].

Urinary albumin concentrations were measured by solid-phase fluorescent immunoassay [12]. Urinary ACR (mg/g) was obtained from the NHANES dataset, calculated by dividing the urinary albumin concentrations by urinary creatinine concentrations and multiplying by 100. Urinary creatinine was measured using a creatininase enzymatic reaction and a Roche Cobas 6000 Analyzer (Indianapolis, IN, USA).

### 2.3. Exposure Measurements

Whole-blood Mn concentrations were measured by quadrupole inductively coupled plasma mass spectrometry [11]. Participants provided urine samples in the mobile examination center; these were analyzed using mass spectrometry for 2013–2014 and 2015–2016. The LLOD was 0.99 µg/L for blood Mn and 0.130 µg/L for urine Mn. For analytes with analytic results below the LLOD, we imputed a value of the LLOD divided by the square root of 2 (LLOD/sqrt [2]). To adjust for variations in urinary dilution, urinary Mn concentrations were divided directly by urine creatinine measures.

### 2.4. Covariates

Demographic characteristics were taken from the questionnaire data and included age and sex. Race/ethnicity was categorized as Mexican American, other Hispanic, Non-Hispanic White, Non-Hispanic Black, Non-Hispanic Asian, and Other (including multi-race). Family poverty-income ratio (PIR) values were categorized into two groups: low income (PIR < 1) and high income (PIR ≥ 1). Body mass index (BMI) was calculated as weight in kilograms divided by height in meters squared (kg/m^2^) at or above the sex-specific 95th percentile of the U.S. Centers for Disease Control and Prevention BMI-for-age growth charts and classified into 4 categories: underweight (z-score < −2), normal (−2 ≤ z-score < 1), overweight (1 ≤ z-score < 2), and obese (z-score ≥ 2). Covariates were chosen a priori, according to prior literature.

### 2.5. Statistical Analysis

Descriptive statistics of participant characteristics, Mn concentrations, and BP and kidney parameters were calculated. Blood and urine Mn concentrations, as well as ACR, were log_10_-transformed to satisfy assumptions for normality. We used weighted multivariable linear regression models using NHANES sampling weights to evaluate the association between log_10_-transformed blood Mn and BP and kidney parameters adjusted for age, gender, BMI, race/ethnicity, and PIR. We calculated the beta (β) estimate and the corresponding 95% confidence intervals (CI). We also conducted sex- and race/ethnicity-stratified analyses to examine the association between blood Mn and BP and kidney parameters. In secondary analyses, we used weighted multivariable linear regression models using NHANES urine metal sampling weights to calculate the beta estimate and the corresponding 95% CI for the association between creatinine-adjusted urine Mn and BP and kidney parameters adjusted for age, gender, BMI z-score, race/ethnicity, and PIR. Sensitivity analyses using age- and sex-specific blood Mn z-scores were conducted to calculate the beta estimate and corresponding 95% CIs [13]. Additional sensitivity analyses were conducted with three additional eGFR formulae that use serum creatinine to estimate GFR to examine any differences in the estimates (Supplemental Methods). Analyses were conducted using SAS v9.4 (SAS Corporation, Cary, NC, USA).

## 3. Results

After exclusions, our final study population consisted of 1931 adolescents. Table 1 displays the sociodemographic characteristics of the study population. The study population was 50.2% female, and the average age was approximately 15 years. The majority of participants self-identified as Non-Hispanic White (31.3%), followed by Mexican American (20.7%). The mean blood Mn level was 11.2 µg/L (range: 3.0–36.9 µg/L), the geometric mean blood Mn was 1.02 µg/L (range: 0.5–1.6 µg/L), and the mean urine Mn level was 0.2 µg/L (range: 0.1–1.9 µg/L). We observed differences in mean blood Mn levels based on self-identified race/ethnicity. Non-Hispanic Blacks had the lowest average blood Mn (9.4 µg/L) whereas Non-Hispanic Asians had the highest average blood Mn (13.8 µg/L). Females had a slightly higher average blood Mn concentrations (12.0 µg/L) than males (10.4 µg/L).

The mean eGFR was 100.4 mL/min/1.73 m^2^. Fourteen participants had an eGFR less than 60 mL/min/1.73 m^2^. The mean SBP was 109 mmHg, while the mean DBP was 61 mmHg. Adolescent hypertension is defined as BP of 130/80 mmHg or higher. Based on this definition, one participant could be putatively classified as having hypertension.

The associations of blood Mn with BP and kidney parameters are shown in Table 2. The unadjusted models showed a significant relationship between blood Mn with higher eGFR, lower SBP, and higher ACR, but null relationships with DBP and BUN.

After adjusting for age, sex, BMI z-score, race/ethnicity, and PIR, we observed that the associations of interest were not significant. For instance, a log_10_-unit increase in blood Mn (µg/L) was only moderately (*p* = 0.1) associated with a 5.8 mL/min higher eGFR (95% CI: −1.6, 13.2) after covariate adjustment. We conducted additional analyses to examine the effect of each individual covariate on the association and observed that sex had the greatest influence on the results. When stratified by sex, we observed null associations between blood Mn levels and eGFR, SBP, DBP, BUN, and ACR (Table 3).

To investigate the differences of BP and kidney parameters related to blood Mn exposure among different races/ethnicities, Table 4 shows the adjusted results of the association of blood Mn with BP and kidney parameters stratified by race/ethnicity. Among Non-Hispanic Black participants, a 10-fold increase in blood Mn (µg/L) was associated with a 14.5 mL/min higher eGFR (95% CI: 0.4, 28.7). Among those identifying as Other Race, which included multiracial participants, a 10-fold increase in blood Mn was associated with a 28.4 mL/min higher eGFR (95% CI: 6.8, 50.1). We also observed a significant association between blood Mn levels and lower SBP among those identifying as Other Race, wherein a 10-fold increase in blood Mn was associated with a 23.8 mmHg lower SBP (95% CI: −36.1, −11.6). Among those identifying as Other Hispanic, a 10-fold increase in blood Mn was associated with a 0.5 mg/g higher ACR (95% CI: 0.1, 1.0). We observed no significant differences between blood Mn levels, DBP, and BUN when stratified by race/ethnicity.

Sensitivity analyses using age- and sex-specific blood Mn z-scores were conducted to evaluate the associations with BP and kidney parameters. The results using the age- and sex-specific blood Mn z-scores showed that the effect estimates did not differ from those presented in Table 2, which included age and sex as covariates in the main model. Additionally, separate sensitivity analyses were conducted to examine the relationships using three additional equations that use serum creatinine to estimate GFR. We found that the effect estimates derived using varied formulae to calculate eGFR did not differ significantly from the creatinine-based Bedside Schwartz formula (Table S1).

There were 691 participants in our study population who had urine Mn measurements from 2013–2015. The unadjusted and adjusted associations of creatinine-adjusted urine Mn with BP and kidney parameters are shown in Table 5. The unadjusted models showed a significant relationship between creatinine-adjusted urine Mn with higher eGFR, but a null relationship with SBP, DBP, BUN, and ACR. We observed that a 10-fold increase in creatinine-adjusted urine Mn was associated with a 16.4 mL/min higher eGFR (95% CI: 11.1, 21.7) after adjusting for age, sex, BMI z-score, race/ethnicity, and PIR. When examining creatinine-adjusted urine Mn and eGFR stratified by sex and race/ethnicity, we observed the similar significant results for both sexes and all race/ethnicities except for associations among Non-Hispanic Asians (Table S2).

## 4. Discussion

The aim of our study was to investigate the relationship between biomarkers of Mn in blood and urine with BP and kidney parameters in adolescents. After adjusting for covariates, we observed that urine Mn concentrations, but not blood, were associated with higher eGFR in adolescents. Furthermore, we observed significant relationships between blood Mn and higher eGFR among individuals identifying as Non-Hispanic Black and Other Race, and higher ACR among individuals identifying as Other Hispanic. We observed no significant differences when stratified by sex.

Our findings were similar to a recent study in adults 18 years and older, which observed linear relationships between both blood and urinary Mn with higher eGFR [14]. In a prior NHANES study, Mn concentrations did not differ significantly between patients with and without chronic kidney disease [15]. However, another study reported elevated Mn levels in predialysis patients with chronic kidney disease compared to healthy controls [5]. Changes in early filtration may lead to hyperfiltration, and are associated with the beginning states of eventual eGFR decline in some chronic kidney diseases [16,17]. Given that Mn is minimally excreted in urine, we interpret the positive association with eGFR cautiously. The positive association we observed in our study between eGFR and urinary Mn could be explained by reverse causality, which has been observed in previous NHANES studies examining urinary metals [18]. In this case, it is possible that reduced renal function may affect metal excretion, as has been shown in studies of Mn levels in adults with chronic kidney disease [5]. The relationship between blood Mn and higher eGFR observed among Non-Hispanic Black and Other race/ethnicity has not been previously reported to our knowledge. A possible hypothesis for this observation may be differences in the absorption, reabsorption, and excretion functions of the kidney, although further research is needed to better understand and address racial/ethnic disparities in chronic kidney disease as well as potential disproportionate Mn exposure. Non-Hispanic Blacks have a greater prevalence of chronic kidney disease risk factors, are 2 to 4 times more likely than Non-Hispanic Whites to have kidney failure, and the incidence of end-stage renal disease among adolescents is 2 to 3 times higher in Non-Hispanic Blacks than in Non-Hispanic Whites [19,20,21]. Prior studies have reported racial/ethnic differences in urine concentration and flow [22], genetics, including variants of the renal sodium channel [23], differences in renal tubular secretion [24], and differences in proximal and distal tubular sodium reabsorption [25].

The present study also observed that blood Mn was associated with lower SBP in an adjusted model among individuals identifying as Other Race/ethnicity. This is similar to the results of an observational study of 367 male workers who were exposed occupationally, which found that workers with the highest Mn exposure had significantly lower SBP, but no relationships were observed with DBP [26]. Two prior studies reported an inverse relationship between BP and Mn, although one study examined daily intake rather than blood or urine biomarkers of exposure [27,28]. A previous study that used NHANES data from 2011–2014 observed that higher urinary Mn levels in an older study population were significantly inversely associated with both SBP and DBP, which was not observed in our study [29]. Differences in the present study and prior reported findings may be due to study design, population demographics (including age and exposure status), choice of Mn biomarker selected, and method of outcome ascertainment. Additionally, exposure sources may provide pivotal information on differences observed among racial/ethnic groups. Possible exposure sources include inhalation of air, consumption of food and water, consumer products such as infant formula, and occupational exposure, with the primary exposure being oral [30]. In the current study, the possible exposure source for this study population may be diet, which is addressed by the NHANES study.

Overall, the mean blood Mn concentrations were higher for female participants than male participants. This is similar to a prior NHANES study that reported that female sex, among participants aged 1–80 years, was associated with higher blood Mn levels [31]. However, we did not observe sex-specific associations between blood Mn with BP and kidney parameters in this study. Examining for potential sex differences is important because prior studies have shown that the ability to cross the blood–brain barrier (which has similar physiology to the glomerular filtration barrier) may vary by sex to influence the timing of puberty [32]. Specifically, early-life Mn accumulation in the hypothalamus may be harmful by stimulating early pubescent development [32]. These differences between the sexes may be due to differences in metabolism of Mn, excretion of Mn from the body, and absorption of Mn via different routes of exposure all of which would indirectly affect relationship with BP and kidney parameters. Additionally, among individuals with chronic kidney disease, Mn levels often decline, and trace element metabolism and excretion are disrupted by chronic kidney disease status [33]. In the present study, we examined healthy adolescents who did not have clinically diagnosed chronic kidney disease; however, this study demonstrates the importance of understanding the relationship between Mn and BP and kidney parameters prior to clinical chronic kidney disease diagnosis.

The selection of blood or urine as a biomarker of Mn exposure is an important consideration. One factor that limits the usefulness of measuring Mn in the blood, urine, or feces as a measure of excess Mn exposure is the relatively rapid rate of Mn clearance from the body. Mn has a blood half-life of 10–42 days, a tissue half-life of 5–7 days, and only 0.1–3% of Mn is excreted in the urine [34,35]. In an animal study, ~50% of the injected dose of Mn chloride in rats was excreted in feces within one day and 85% by the 23rd day, suggesting that biliary excretion is the main route of clearance [36,37]. Recently, a study among welders reported that toenail measures were a valid proxy of cumulative Mn exposure 7–12 months earlier [38]. However, for practical reasons, blood Mn is used as a biomarker of exposure in many epidemiological studies since a standard biomarker for measuring Mn has not yet been established. Studies are needed to examine multiple media exposures of Mn and associations with BP and kidney parameters, as well as the joint effects of Mn, other metals, and nephrotoxicants.

One limitation of this study is its cross-sectional design. Interpretation of findings is limited in cross-sectional studies where temporality and therefore directionality cannot be determined. We emphasize that reverse causality cannot be ruled out, meaning that it cannot be definitively dismissed that the temporality of the exposure–outcome process is reversed i.e., alterations in eGFR affect blood Mn levels. Since this study only includes healthy adolescents in the study population, we did not examine associations in individuals with clinical chronic kidney disease; therefore, this is an exploratory cross-sectional study and examination of clinical endpoints or prognosis was not the intended outcome. Additionally, outcome measures in this study were collected at a single time point, and longitudinal assessment of eGFR, BP, BUN, and ACR will enhance future studies. Another limitation of this study was the use of the eGFR calculated with the Bedside Schwartz formula. This equation uses a single constant, independent of sex and age, as well as serum creatinine concentration, which is available in basic metabolic panels. Although debated, this formula may produce biased estimates, and may not be accurate for estimating GFR in adolescents since the formula was based on children aged 1 to 16 years [39,40]. Furthermore, sensitivity analyses using three formulae for eGFR produced similar effect estimates. We acknowledge that it would be preferable to analyze eGFR derived from cystatin C as this biomarker is less sensitive to differences in BMI, muscle mass, and diet. There are many formulae used to calculate eGFR, including a recently published estimating equation using cystatin C and age- and sex-specific constants for eGFR, to yield less biased estimates [41]. The considerations of which formulae should be used are not uniform and the debate remains open regarding the preferred equations for estimating GFR depending on the molecular laboratory analyses and the population being studied.

Our study also has notable strengths. This is a large study investigating the relationship of blood and urine Mn with BP and kidney parameters among U.S. adolescents. We were able to utilize data from a large sample of U.S. adolescents to ensure statistical efficiency. The sample population encompasses a diverse selection of healthy U.S. adolescents. We studied a large, representative sample of the national population, as the NHANES survey is designed to accurately represent the U.S. population. We applied sampling weights to account for NHANES oversampling among certain populations (i.e., Hispanics, Non-Hispanic Blacks, and individuals below the poverty guidelines). Thus, our findings may have enhanced generalizability to the U.S. adolescent population, an improvement over studies of adolescents in smaller, regional areas. This study provides an important foundation from which to design prospective studies investigating the relationship of developmental Mn exposure, BP, and renal health in other studies and populations.

## 5. Conclusions

In conclusion, this cross-sectional study using NHANES data identified no relationships between blood Mn concentrations in healthy U.S. adolescents and BP or kidney parameters. We observed a putative positive relationship between urine Mn and eGFR. Longitudinal studies are needed to further examine these exploratory findings of the complex relationships between Mn in kidney parameters and BP regulation, including race/ethnicity- and sex-related differences.

## Figures and Tables

**Table 1:** Study population demographic characteristics, NHANES, 2013–2018 (*n* = 1931).

	N	%
Sex		
Male	962	49.8
Female	969	50.2
Race/Ethnicity		
Mexican American	399	20.7
Other Hispanic	181	9.4
Non-Hispanic White	604	31.3
Non-Hispanic Black	386	20.0
Non-Hispanic Asian	209	10.8
Other Race	152	7.9
Family Poverty-Income Ratio		
<1 (low)	570	29.6
≥1 (high)	1361	70.4
Body Mass Index		
Underweight (z-score < −2)	67	3.5
Normal (−2 ≤ z-score < 1)	1081	56.0
Overweight (1 ≤ z-score < 2)	337	17.4
Obese (z-score ≥ 2)	446	23.1
	Mean ± Standard Deviation	
Age (years)	15.4 ± 2.2	
Blood Mn (µg/L)	11.2 ± 3.9	
Urine Mn (µg/L)	0.15 ± 0.17	
eGFR (mL/min/1.73 m^2^)	100.4 ± 21.6	
SBP (mmHg)	108.6 ± 9.6	
DBP (mmHg)	60.7 ± 10.3	
BUN (mg/dL)	11.4 ± 3.5	
ACR (mg/g)	24.1 ± 89.2	

Mn: Manganese; eGFR: estimated glomerular filtration rate; SBP: systolic blood pressure; DBP: diastolic blood pressure; BUN: blood urea nitrogen; ACR: albumin creatinine ratio.

**Table 2:** Unadjusted and adjusted associations of blood Mn (log_10_ µg/L) and BP and kidney parameters using survey weighted linear regression.

	Unadjusted			Adjusted ^1^		
	β	95% CI	*p*-Value	β	95% CI	*p*-Value
eGFR	23.2	(14.4, 32.0)	<0.0001	5.8	(−1.6, 13.2)	0.1
SBP	−3.8	(−7.3, −0.4)	0.03	0.4	(−3.0, 3.8)	0.8
DBP	−0.9	(−5.6, 3.8)	0.7	−1.3	(−6.0, 3.4)	0.6
BUN	−1.3	(−3.3, 0.6)	0.2	−0.03	(−1.7, 1.6)	0.9
ACR	0.2	(0.02, 0.4)	0.03	0.04	(−0.2, 0.2)	0.7

^1^ Models were adjusted for age, sex, BMI z-score, race/ethnicity, and PIR. CI: confidence interval; eGFR: estimated glomerular filtration rate; SBP: systolic blood pressure; DBP: diastolic blood pressure; BUN: blood urea nitrogen; ACR: albumin creatinine ratio.

**Table 3:** Adjusted ^1^ associations of blood Mn (log_10_ µg/L) with BP and kidney parameters using survey weighted linear regression, stratified by sex.

	Males			Females		
	β	95% CI	*p*-Value	β	95% CI	*p*-Value
eGFR	5.7	(−5.9, 17.3)	0.3	3.2	(−8.0, 14.4)	0.6
SBP	−0.6	(−6.5, 5.3)	0.8	1.4	(−3.1, 6.0)	0.5
DBP	0.6	(−8.2, 9.3)	0.9	−2.4	(−8.6, 3.8)	0.4
BUN	−0.7	(−3.0, 1.5)	0.5	0.7	(−1.4, 2.8)	0.5
ACR	0.2	(−0.2, 0.5)	0.3	−0.08	(−0.3, 0.2)	0.5

^1^ Models were adjusted for age, BMI z-score, race/ethnicity, and PIR. CI: confidence interval; eGFR: estimated glomerular filtration rate; SBP: systolic blood pressure; DBP: diastolic blood pressure; BUN: blood urea nitrogen; ACR: albumin creatinine ratio.

**Table 4:** Adjusted ^1^ associations of blood Mn (log_10_ µg/L) with BP and kidney parameters using survey weighted linear regression, stratified by race/ethnicity.

	eGFR			SBP			DBP			BUN			ACR		
	β	95% CI	*p*-Value	β	95% CI	*p*-Value	β	95% CI	*p*-Value	β	95% CI	*p*-Value	β	95% CI	*p*-Value
Mexican American	5.7	(−8.9, 20.4)	0.4	−1.05	(−9.7, 7.6)	0.8	4.52	(−5.4, 14.5)	0.4	−0.47	(−2.7, 1.8)	0.7	−0.2	(−0.6, 0.3)	0.4
Other Hispanic	29.4	(−0.4, 59.3)	0.053	−3.3	(−13.0, 6.4)	0.5	6.37	(−6.5, 19.2)	0.3	2.1	(−2.6, 6.9)	0.4	0.5	(0.1, 1.0)	0.01
Non-Hispanic White	−1.99	(−12.8, 8.9)	0.7	2.4	(−2.9, 7.7)	0.4	−1.82	(−8.6, 5.0)	0.5	0.5	(−2.0, 3.0)	0.7	0.03	(−0.3, 0.4)	0.8
Non-Hispanic Black	14.5	(0.4, 28.7)	0.04	5.9	(−1.6, 13.4)	0.1	−4.76	(−13.0, 3.6)	0.2	−1.03	(−3.6, 1.7)	0.4	0.1	(−0.2, 0.4)	0.3
Non-Hispanic Asian	15.9	(−33.6, 65.4)	0.5	5.2	(−2.5, 13.0)	0.2	−3.51	(−14.7, 7.7)	0.5	−2.3	(−6.4, 1.9)	0.3	−0.1	(−0.5, 0.3)	0.6
Other Race	28.4	(6.8, 50.1)	0.01	−23.8	(−36.1, −11.6)	0.0003	−8.37	(−26.9, 10.2)	0.4	0.02	(−3.1, 3.1)	0.9	−0.2	(−0.7, 0.3)	0.5

^1^ Models were adjusted for age, sex, BMI z-score, and PIR. CI: confidence interval; eGFR: estimated glomerular filtration rate; SBP: systolic blood pressure; DBP: diastolic blood pressure; BUN: blood urea nitrogen; ACR: albumin creatinine ratio.

**Table 5:** Unadjusted and adjusted associations of urine Mn (log_10_ µg Mn/g creatinine) with BP and kidney parameters using survey weighted linear regression. Urine Mn levels were creatinine-adjusted in all models.

	Unadjusted			Adjusted ^1^		
	β	95% CI	*p*-Value	β	95% CI	*p*-Value
eGFR	19.5	(14.4, 24.6)	<0.0001	16.4	(11.1, 21.7)	<0.0001
SBP	−1.6	(−4.0, 0.7)	0.2	0.1	(−2.1, 2.3)	0.9
DBP	1.7	(−1.4, 1.5)	0.3	0.4	(−2.3, 3.1)	0.8
BUN	−0.6	(−1.5, 0.4)	0.2	−0.5	(−1.3, 0.4)	0.3
ACR	0.07	(−0.04, 0.2)	0.2	−0.02	(−0.1, 0.1)	0.8

^1^ Models were adjusted for age, sex, BMI z-score, race/ethnicity, and PIR. CI: confidence interval; eGFR: estimated glomerular filtration rate; SBP: systolic blood pressure; DBP: diastolic blood pressure; BUN: blood urea nitrogen; ACR: albumin creatinine ratio.

## Data Availability

Publicly available datasets were analyzed in this study. The data can be found here: https://wwwn.cdc.gov/nchs/nhanes/ (accessed on 1 July 2021).

## Supplementary Materials

The following are available online at https://www.mdpi.com/article/10.3390/children8100846/s1, Table S1: Comparison of adjusted ^1^ effect estimates of blood Mn (log10 µg/L) concentrations with eGFR using four serum-creatinine based eGFR formulae, and stratified by sex or race/ethnicity, Table S2: Adjusted associations of urine Mn (log10 µg Mn/g creatinine) and eGFR using survey weighted linear regression, stratified by sex or race/ethnicity. Urine Mn levels were creatinine-adjusted in all models.
