# Supplementary material for: Association of Manganese Biomarker Concentrations with Blood Pressure and Kidney Parameters among Healthy Adolescents: NHANES 2013–2018
Source: Children (Basel). 2021 Sep 25;8(10):846. doi: 10.3390/children8100846 (PMC8534392; doi:10.3390/children8100846)
Supplement: Supplementary file 1 [file children-08-00846-s001.zip › children-1340702-supplementary.pdf]

1. Supplemental Methods

1.1. Outcome Measurements

In a sensitivity analysis, we examined the relationships using three additional equations that use serum creatinine to estimate GFR. The original Bedside-Schwartz formula used to calculate eGFR and used as the main method of measuring eGFR was calculated using the following formula:

$$\text{eGFR} = 0.413 \times \frac{\text{Height (cm)}}{\text{Serum Creatinine (mg/dL)}} \quad (1)$$

as recommended in conjunction with this laboratory method [1]. The CKiD under 25 eGFR measurement was calculated using the following formula:

$$\text{eGFR} = \text{K (age- and sex-dependent constant)} \times \frac{\text{Height (cm)}}{\text{Serum Creatinine (mg/dL)}} \quad (2)$$

as recently recommended in conjunction with this laboratory method [2]. This calculation takes into account age and sex of individuals up until age 25 years. The Gao quadratic eGFR equation was calculated using the following formula:

$$\text{eGFR} = 0.68 \times \frac{\text{Height (cm)}}{\text{Serum Creatinine (mg/dL)}} - 0.0008 \times \left(\frac{\text{Height (cm)}}{\text{Serum Creatinine }(\frac{\text{mg}}{\text{dL}})}\right)^2 + 0.48 \times \text{age} - (21.53 \text{ for males and } 25.68 \text{ for females}) \quad (3)$$

as recommended in conjunction with this laboratory method [3]. The Full Age Spectrum eGFR equation was calculated using the following formula:

$$\text{eGFR} = \frac{107.3}{\frac{\text{Serum Creatinine }(\frac{mg}{dL})}{Q}} \quad (4)$$

where

$$Q = 3.94 - (13.4 \times \text{height}) + (17.6 \times \text{height}^2) - (9.84 \times \text{height}^3) + (2.04 \times \text{height}^4) \quad (5)$$

as recently recommended in conjunction with this laboratory method [4,5].

Table S1. Comparison of adjusted[1] effect estimates of blood Mn ($\log_{10}$ µg/L) concentrations with eGFR using four serum-creatinine based eGFR formulae, and stratified by sex or race/ethnicity.

	**Creatinine-Based Bedside Schwartz eGFR equation**			**CKiD under 25 eGFR equation**			**Gao Quadratic eGFR equation**			**Full Age Spectrum eGFR equation**		
	**β**	**95% CI**	**p-value**	**β**	**95% CI**	**p-value**	**β**	**95% CI**	**p-value**	**β**	**95% CI**	**p-value**
**Main Model** **Unadjusted**	23.2	(14.4, 32.0)	<0.0001	9.56	(1.9, 17.2)	0.01	12.5	(7.3, 17.7)	<0.0001	14.9	(5.0, 24.8)	0.004
**Adjusted**	5.8	(-1.6, 13.2)	0.1	5.9	(-1.4, 13.3)	0.1	3.9	(-0.7, 8.4)	0.1	6.8	(-2.5, 16.2)	0.1
**Sex**												
Females	3.2	(-8.0, 14.4)	0.6	3.1	(-7.4, 13.7)	0.5	2.0	(-5.4, 9.4)	0.6	4.9	(-9.0, 18.9)	0.5
Males	5.7	(-5.9, 17.3)	0.3	7.6	(-4.9, 20.0)	0.2	3.4	(-3.7, 10.5)	0.3	7.0	(-5.1, 19.2)	0.2
**Race/Ethnicity**												
Mexican American	5.7	(-8.9, 20.4)	0.4	7.4	(-8.3, 23.1)	0.3	5.6	(-4.7, 15.9)	0.3	8.8	(-8.5, 26.2)	0.3

Other Hispanic	29.4	(-0.4, 59.3)	0.053	26.5	(-4.3, 57.3)	0.09	20.6	(5.9, 35.4)	0.01	21.7	(-8.0, 51.3)	0.1
Non-Hispanic White	-1.99	(-12.8, 8.9)	0.7	-1.8	(-12.0, 8.4)	0.7	-1.5	(-8.9, 5.9)	0.7	1.7	(-11.8, 15.2)	0.8
Non-Hispanic Black	14.5	(0.4, 28.7)	0.04	14.3	(1.0, 27.7)	0.03	11.0	(2.0, 20.0)	0.02	17.8	(-2.9, 38.4)	0.08
Non-Hispanic Asian	15.9	(-33.6, 65.4)	0.5	14.0	(-33.3, 61.2)	0.5	-1.5	(-18.0, 14.9)	0.8	1.9	(-51.9, 55.8)	0.9
Other Race	28.4	(6.8, 50.1)	0.01	27.3	(6.7, 47.8)	0.01	19.1	(4.9, 33.2)	0.01	21.3	(0.001, 42.6)	0.05

[1] Models were adjusted for age, sex, BMI z-score, race/ethnicity, and PIR.
CI: confidence interval; eGFR: estimated glomerular filtration rate

Table S2. Adjusted associations of urine Mn ($\log_{10}$ µg Mn/g creatinine) and eGFR using survey weighted linear regression, stratified by sex or race/ethnicity. Urine Mn levels were creatinine-adjusted in all models.

	**eGFR**		
	**β**	**95% CI**	**p-value**
**Sex[1]**			
Females	15.4	(7.9, 22.9)	0.0002
Males	15.4	(8.6, 22.3)	<.0001
**Race/Ethnicity[2]**			
Mexican American	10.9	(1.8, 20.0)	0.02
Other Hispanic	35.8	(23.4, 48.2)	<.0001
Non-Hispanic White	14.7	(6.2, 23.2)	0.0014
Non-Hispanic Black	17.4	(8.3, 26.6)	0.0005
Non-Hispanic Asian	7.7	(-6.3, 21.7)	0.3
Other Race	20.9	(7.3, 34.6)	0.004

[1] Models were adjusted for BMI z-score, race/ethnicity, and PIR.
[2] Models were adjusted for sex, BMI z-score, and PIR.
CI: confidence interval; eGFR: estimated glomerular filtration rate

References

1. Schwartz, G.J.; Muñoz, A.; Schneider, M.F.; Mak, R.H.; Kaskel, F.; Warady, B.A.; Furth, S.L. New equations to estimate GFR in children with CKD. *J Am Soc Nephrol* **2009**, *20*, 629-637, doi:10.1681/asn.2008030287.
2. Pierce, C.B.; Muñoz, A.; Ng, D.K.; Warady, B.A.; Furth, S.L.; Schwartz, G.J. Age- and sex-dependent clinical equations to estimate glomerular filtration rates in children and young adults with chronic kidney disease. *Kidney Int* **2021**, *99*, 948-956, doi:10.1016/j.kint.2020.10.047.
3. Gao, A.; Cachat, F.; Faouzi, M.; Bardy, D.; Mosig, D.; Meyrat, B.J.; Girardin, E.; Chehade, H. Comparison of the glomerular filtration rate in children by the new revised Schwartz formula and a new generalized formula. *Kidney Int* **2013**, *83*, 524-530, doi:10.1038/ki.2012.388.
4. Hoste, L.; Dubourg, L.; Selistre, L.; De Souza, V.C.; Ranchin, B.; Hadj-Aïssa, A.; Cochat, P.; Martens, F.; Pottel, H. A new equation to estimate the glomerular filtration rate in children, adolescents and young adults. *Nephrol Dial Transplant* **2014**, *29*, 1082-1091, doi:10.1093/ndt/gft277.
5. Pottel, H.; Hoste, L.; Dubourg, L.; Ebert, N.; Schaeffner, E.; Eriksen, B.O.; Melsom, T.; Lamb, E.J.; Rule, A.D.; Turner, S.T.; et al. An estimated glomerular filtration rate equation for the full age spectrum. *Nephrol Dial Transplant* **2016**, *31*, 798-806, doi:10.1093/ndt/gfv454.
